# A Good Method of Cooking Frozen Meat

**Published:** 1912-04-27

**Authors:** 


### INSTITUTIONAL HOUSEKEEPING.

### A Good Method of Cooking Frozen Meat.

In many institutions frozen meat is used exclusively and with varying degrees of satisfaction to those for whom it is provided. Difference in cost is the reason for its use, and when special care is given to the quality and to the cooking a considerable saving can be effected. If foreign meat be used it is essential that only a superior quality should be purchased, and strict supervision should be exercised in regard to the condition on receipt. It should be hung in a well ventilated larder for at least twelve hours before cooking, and during cold weather it is better to have the meat hanging for twenty-four hours in a temperate larder. It should then be brought into a warm kitchen for two hours before cooking. These precautions will prevent the disappointment of finding the apparently well cooked joint much too underdone inside. The joints should be thoroughly wiped with a clean linen cloth and any unsightly edges trimmed off. By far the most satisfactory way to cook foreign meat (and, indeed, any meat) is in a double cooker, allowing about three-quarters of an hour to brown the joints in the oven afterwards. In the patent cooker no water is used; hence all the juices of the meat are retained, the flavour is preserved, and the loss in weight is considerably less than by any other method.

The initial cost of the cookers is somewhat large, and at first when this method is adopted the co-operation of the cook must be won. After a fair trial, however, a sensible cook will soon see the advantage of the method. Having well started the meat in the double vessels, it requires almost no attention for a considerable time, thus her hands are more free for the forwarding of other portions of the work. About three-quarters of an hour before the meat is required it must be taken from the cooker, well dredged with flour, basted, and put in a hot oven to brown. It must be well basted from time to time until finished.

Double vessels (Captain Warren's pattern) large enough to contain four legs of mutton or three sirloins of beef—30 lb. to 35 lb.—cost between £3 and £4. This amount, however, will be saved on the meat bill in a few months at most, and the improvement in flavour, tenderness, and appearance of the joints will for ever remove any prejudice against frozen meat.

When cold meat is to be served an effort should be made to arrange the ordering so that the joints will not be cut while hot. Two smaller joints, one for eating while hot, and one for eating cold, will give much more satisfaction than one large joint served twice.

When frozen meat is to be used in an institution it should be used throughout for all members of the staff as well as for patients. The saving should not be attempted for one section of the household only.

### HOUSEKEEPING QUERIES.

### To Our Readers.

In this column questions are dealt with relating to cookery and household recipes, the duties of the domestic staff, estimates of quantities required for given numbers, and the cost of diet for patients and staff. Arrangements have been made to deal with samples submitted for examination and report on them.

### To Keep Milk Sweet.

"Temporary Matron": It is unfortunate that the milk for the wards cannot be kept apart from other foods, but one point is *imperative*—strong-smelling and flavoured foods must not be in the dairy, which is perhaps also used as larder—cheese, fish, onions, apples, etc., will ruin milk. Vessels may be of tin, earthenware, or enamel; the main thing is their *absolute cleanliness*—separate brush for scrubbing them, and separate cloths are essential. Boiling water must be used, not merely hot water, and a little soda to remove greasiness of the milk-fat. Double sets of vessels are desirable in order that one set can be put out in the air and sun to sweeten daily. Lids must not be used for the pans, but they should be covered with hoops, over which fine muslin is stretched, or merely pieces of muslin can be laid across, or even sheets of perforated paper. The covers must admit air, but not dust. In very warm weather, when the milk is to be heated, a tiny pinch of bicarbonate of soda can be added. This lessens the risk of it curdling.

### How to Use Gifts of Old Table-linen.

"Frugal": If your bundles contain any good damask they can be turned to very good account even if much worn. A tablecloth usually wears along the seams, and worn places of this kind may be darned with flax thread and last a long time. If the general fabric is not worn but holes have been torn through carelessness in the laundry, these may be deftly patched with old damask matching as far as possible. The patch should be cut three-quarters of an inch larger than the hole, and darned in. When the damask is beyond use as a tablecloth, however, the best parts may be used for tray-cloths, for serviettes, or for little cloths to lay under fish or under pie-dishes. And the worst pieces will make excellent polishing rags.

### To Prevent Iron-mould on Linen.

"L. M."—Examine whether the copper, or the boiler-stick with which the clothes are stirred while boiling, or the zinc baths, or the clothes-pegs be rusty. The boiler-stick and pegs should be made of plain wood, not tin-bound, as then rust quickly forms. Note also if there are any iron nails used in the manufacture of the wooden tubs or boiler-lid, as these are a common cause of rust.

### How to Use "Scotch Hands."

"Lancashire."—Before use "Scotch hands" must always be dipped into boiling water to scald them, then rubbed with a little salt, and laid for at least ten minutes in cold water containing salt. Unless this is done the butter invariably sticks, and will be tiresome to shape.

### Cutting and Serving Puddings.

"Matron of Cottage Hospital."—To cut puddings, etc., neatly into squares get your ironmonger to supply some flat strong pieces of tin, about five inches long and four inches broad, with one end rolled up to form a handle. The edge cuts the puddings easily and the slabs can be quickly and neatly raised—an impossibility with a curved surface.

